# Challenges in Documenting Non-Fatal Drowning Disability in Bangladesh: A Community-Based Survey

**DOI:** 10.3390/ijerph18189738

**Published:** 2021-09-15

**Authors:** Jagnoor Jagnoor, Medhavi Gupta, Aliki Christou, Rebecca Q. Ivers, Soumyadeep Bhaumik, Kamran Ul Baset, Kris Rogers, Aminur Rahman

**Affiliations:** 1 The George Institute for Global Health India, New Delhi 110025, India; sbhaumik@georgeinstitute.org.in; 2 The George Institute for Global Health, Newtown, NSW 2042, Australia; mgupta@georgeinstitute.org.au (M.G.); krogers@georgeinstitute.org (K.R.); 3 School of Population Health, Faculty of Medicine and Health, The University of Sydney, Sydney, NSW 2042, Australia; aliki.christou@sydney.edu.au; 4 School of Public Health and Community Medicine, University of New South Wales, Sydney, NSW 2042, Australia; rebecca.ivers@unsw.edu.au; 5 Centre for Injury Prevention and Research, Dhaka 1206, Bangladesh; kamran_baset@yahoo.co.uk (K.U.B.); aminur@ciprb.org (A.R.)

**Keywords:** drowning, non-fatal drowning, disability, morbidity, low-and middle-income country, household survey

## Abstract

Limited access to health care and the lack of robust data systems means non-fatal drownings are largely missed in low-and middle-income countries. We report morbidity among individuals who experienced non-fatal drowning in the Barishal Division, Bangladesh. A representative household survey was conducted in the Barishal Division in southern Bangladesh between September 2016 and February 2017, covering a population of 386,016. The burden of non-fatal drowning was assessed using the WHODAS 2.0 disability assessment tool, a generic assessment instrument for health and disability. A total of 5164 non-fatal drowning events occurred in the one year preceding the survey. Among these 18% were multiple events. From these, 4235 people were administered the WHODAS 2.0 questionnaire. Non-fatal drowning incidence rates were highest in children aged 1–4 years at 5810 per 100,000 population, and among males. Non-fatal drowning was associated with lower socio-economic status and larger family sizes. Few respondents (6.5%; 95% CI: 4.5–8.4%) reported some level of disability (WHODAS-12 score > 8). Incidence of non-fatal drowning is high in the population, however limited impact on morbidity was found. There is a need to develop tools and methodologies for reliable and comparable data for non-fatal drowning, especially to capture post-event disability in children.

## 1. Introduction

Drowning is a major cause of mortality and morbidity in children and adults, with over 90% of drowning occurring in low-and middle-income countries (LMICs) [1]. In Bangladesh, drowning accounts for 43% of deaths of children aged 1–5 years and is currently the leading cause of death in this age group [2]. In 2005, the rate of non-fatal drowning in children aged 1–17 years old in Bangladesh was 118 per 100,000 population, several times higher than the rate of drowning deaths (28.6/100,000) [2]. Advancements in verbal autopsy methods have led to reasonable measurements of the extent of the burden of fatal drowning [3], however morbidity or disability, such as brain and respiratory injury, resulting from non-fatal drowning incidents has not been adequately explored or quantified in LMICs.

Non-fatal drowning can have many long-term health, social and economic consequences including permanent neurological disability that requires ongoing care, often with catastrophic health expenditure for families [4,5,6]. In 2000, it was estimated that over 1.3 million years were lost to premature death or disability from drowning or near drowning and that over 60% of disability-adjusted life years (DALYs) lost to drowning and non-fatal drowning were among children aged under 15 years [7]. More recent data from 2019 suggests the burden from non-fatal drowning has increased, where drowning accounted for over 13 million DALYs [8] and was among the top ten causes of years of life lost due to disability among 10–24 year olds globally [9]. Across both LMIC and high-income country (HIC) contexts, there is a complete absence of data in the literature documenting the burden of disability resulting from drowning related injuries, yet estimations based on these limited data suggest that for every fatal drowning event there are between one and four non-fatal drowning events serious enough to require hospitalisation [4,10]. Follow-up studies of children admitted to paediatric intensive care following a drowning incident showed long term cognitive and neurological deficits [11]. Understanding how this contributes to disability and impacts the quality of life in children and adults in low-income countries is needed. The lack of such data and absence of any reporting mechanism prevents an accurate assessment of the burden of serious injuries and lifelong disability arising from drowning, and also means that the incidence and consequences of non-fatal drowning events are likely to be seriously underestimated.

The recommended Utstein style of classification of drowning morbidity outcomes as either moderately disabled, severely disabled, vegetative state/coma or brain death [12], is one suggested measure of the impact of drowning on functioning, however this is challenging to implement in community-based measures of disability, and also does not give a full picture on the impact of disability on the social and functional aspects of an individual’s life. In 2015, the World Health Organization (WHO) recommended that countries make efforts to improve data on drowning related mortality and morbidity, including a better capture of data for non-fatal drowning [1]. This is particularly important for assessing the impact of drowning prevention interventions on both mortality and morbidity outcomes, as well as the need for rehabilitation programs. The absence of a clear definition for non-fatal drowning and its resultant outcomes has also been a barrier to documenting the burden and impact of these morbidities. Disability refers to difficulties in three main areas of human functioning—impairments (in body function or alterations in body structure), activity limitations (difficulties in executing activities such as walking or eating) and participation restrictions (difficulties in engaging in any area of life such as facing discrimination in employment, transportation) [13].

There have been no studies assessing functional outcomes after drowning and the long-term consequences associated with non-fatal drowning in low-income settings. Moreover, there are currently no data collection tools validated for the specific assessment of drowning morbidity at a population level. Various methods and instruments have been developed and used to measure disability in populations [9,13]. The World Health Organization Disability Assessment Schedule (WHODAS) is one such method developed to identify consequences for any disorder that might have an impact on everyday functioning [14]. It provides a standardised way to measure health and disability across cultures.

The aim of this study was to assess the impact of non-fatal drowning on disability and functioning using the WHODAS 2.0 disability assessment tool among individuals who experienced a non-fatal drowning in the Barishal Division in Bangladesh. In addition, we describe factors associated with non-fatal drowning incidents.

## 2. Materials and Methods

### 2.1. Study Design and Setting

The study was carried out in the southern Barishal Division of Bangladesh. Barishal has a total population of over 8 million and is one of the most vulnerable areas to disaster and climate change in Bangladesh; all of the country’s six districts are affected by water related hazards and disasters. To measure the burden of drowning, a population-based, cross-sectional household survey using a multi-stage stratified sample was conducted in all six districts of the Barishal Division (Barguna, Barishal, Bhola, Jhalokathi, Patuakhali and Pirojpur) between September 2016 and February 2017. Detailed methodology for the cross-sectional survey and results on the prevalence of fatal drowning has been previously published [15].

### 2.2. Procedures

Data collectors were trained based on the training manual Measuring Health and Disability Manual for WHO Disability Assessment Schedule WHODAS 2.0 [16]. Trained data collectors used pre-tested structured questionnaires to collect information through face-to-face interviews. The interviewee was the person who experienced the drowning event (if they were an adult over the age of 18 years), or household member above 18 years if the survivor was not available. Where the drowning event survivor was a child, their caregiver answered the questionnaire. An electronic data capture system, REDCap application [17], was used on tablets for data collection. To ensure the reliability of data, 2% of the sample was re-surveyed.

All participants (or their caregivers if under the age of 18) provided written informed consent before participating in the study.

### 2.3. Measures

The baseline survey collected data on household socio-demographic characteristics, all-cause mortality and all cause injury mortality, care-seeking behaviours following injury events, as well as community knowledge, attitudes and practices related to drowning and drowning prevention, and disaster preparedness. Injury was defined in accordance with the International Classification of Disease (ICD), Version 10, Chapter XX recording intent and mechanism of injury [18]. The operational definition of injury used was “any external harm resulting from an assault, fall, burn, mechanical injury, poisoning, transportation, suffocation, or drowning related event resulting in the loss of one or more days of normal daily activities, school, or work [19]. Drowning was described as the process of experiencing respiratory impairment from submersion or immersion in liquid [20] while non-fatal drowning was defined as any event with submersion of face in water associated with breathing difficulty, which required assistance for recovery from water and/or first response”. Information on all non-fatal drowning injuries was collected over a one-year recall period for up to four non-fatal events. The WHODAS 2.0 [14] 12-item survey was administered to each individual identified as having experienced a non-fatal drowning event. Participants were asked to complete the survey for the most recent non-fatal drowning event. The recall period used was 30 days, as per the for WHODAS tool. The validated Bangla translated version of the tool was used in this study and the study was registered for non-commercial use with WHODAS (13/08/16).

The World Health Organization Disability Assessment, WHODAS 2.0-12 proxy administered comprises of 12 items, grouped in six domains: (a) cognition; (b) mobility; (c) self-care; (d) getting along with people; (e) life activities subdivided into household activities and work/school activities; and (f) participation in society [16]. For each item, respondents rate the level of difficulty experienced on a five-point scale from “none” to “extreme or cannot do”. An overall disability score was calculated ranging from 0 (no disability) to 48 (maximum disability). Disability level was categorised according to scores as no disability (score 0–7) or any disability (score 8–48). This tool is validated to be used across all causes of morbidity.

The tool has strong validity, reliability, and cross-cultural applicability, including in Bangladesh. It is also appropriate for population level implementation and both the tool and its proxy have been validated in Bangla and for use across groups with varying backgrounds and training [15,16]. We also consulted experts from WHO in the field of drowning and health related quality of life and disability, as well respiratory physicians for selection of the tool. They recommended replacing questions related to “work” with “school or play” for children, to make the questions applicable. However, the tool has not been specifically validated for drowning-related morbidity.

### 2.4. Data Analysis

Weights were created for the survey data to appropriately adjust for differences in the probability of selection and response rates according to age and sex. We calculated the probability of selection based on sampling information (number of upazilas, number of villages), and then adjusted this to the age and sex distribution from the 2011 Bangladesh Population and Housing Census. All data were analysed using SAS 9.4 with SAS/STAT 14.2 (SAS Institute, Cary, NC, USA).

Means of continuous variables (such as WHODAS score and SES Score) and rates (/100,000 for fatal and near-drowning, percentage for other variables) were estimated using Taylor Series method in the survey procedure. This incorporated the survey weights and the other features of survey design (stratification by district, and cluster sampling of villages). We used survey regression procedures similarly when estimating regression models for both continuous outcomes and binary outcomes (survey logistic regression).

## 3. Results

A total of 95,124 households were visited, of which 92,616 participated in the baseline survey (Response rate 98%) covering a total population of 386,016. A total of 5164 non-fatal drowning events occurred in the one year preceding the survey. Among these, 18% of respondents reported experiencing multiple non-fatal drowning events, and 4235 were administered the WHODAS 2.0 questionnaire (Table 1). The mean number of days between the drowning event and data collection was 165 (95% CI: 135–196).

As shown in Table 2, non-fatal drowning incident rates were highest in children aged 1–4 years, followed by those aged 5–9 years, and rates among males were higher overall. Among adolescents, males experienced non-fatal drowning almost twice as frequently as females. Non-fatal drowning rates were also almost twice as high in rural areas compared to urban areas.

### 3.1. Recovery from Non-Fatal Drowning

Unconsciousness was experienced by 8.4% (*n* = 356) of respondents following recovery from water. Most survivors could walk without assistance (66%, *n* = 2795) but 27.1% (*n* = 1143) needed assistance to walk, and 6.8% (*n* = 288) could not walk.

Table 3 shows the disability summary scores for non-fatal drowning events. Most respondents (93.4%, *n* = 3955) reported no disability as a result of the drowning incident, while some level of disability (WHODAS-12 score > 8) was reported for 6.5% (*n* = 275) (95% CI: 4.5–8.4%) of respondents.

On average, the respondents were completely unable to work or carry out usual activities for approximately two days (Table 3 below).

Table 4 shows the level of disability for each of the 12 items assessed by the WHODAS tool. Of respondents, 13% (*n* = 551) reported mild difficulties standing for long periods and were emotionally affected by their health problem after a drowning event. Almost 10% (*n* = 424) of respondents had difficulties concentrating on a task for ten minutes or walking long distances.

### 3.2. Risk Factors for Non-Fatal Drowning

Table 5 shows the association between various socio-demographic characteristics and non-fatal drowning. Multiple logistic regression analysis for non-fatal drowning showed that children aged 1–4 years had almost 50 times higher, and children aged 5 to 9 years of age had 14 times higher odds of non-fatal drowning compared to infants, after adjusting for sex, socio-economic status, maternal education, and number of children in a household. Higher non-fatal drowning odds were also significantly associated with lower socio-economic status and higher number of children in the household in the multivariable model, with odds reducing with each increasing wealth quintile. There was also a significant association between gender and non-fatal drowning, with females having significantly lower odds compared to males.

Common circumstances surrounding the non-fatal drowning events were also captured. Over half (58%) of events occurred between 12:00 p.m. and 3:59 p.m., and 31% occurred between 8:00 a.m. and 11:59 a.m. (data not shown). Most non-fatal drowning events (56.8%) occurred during the monsoon season, between June and October (data not shown).

### 3.3. Care-Seeking Behaviour for Non-Fatal Drowning

**Figure 1:**
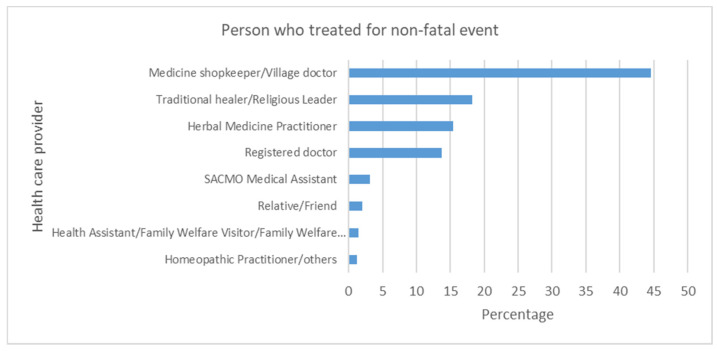
Personnel attending person with non-fatal drowning event.

Health consultation was sought by only 20% (*n* = 847) of respondents. Only a small proportion (5.8%, *n* = 256) of respondents were admitted to the hospital. Surgery was reported for 1.4% (*n* = 59) of respondents. See Figure 1 for a breakdown on personnel who attended post-event.

## 4. Discussion

The survey found a non-fatal drowning rate of 696.7 per 100,000 population across the Barishal Division. Factors associated with non-fatal drowning events, such as younger children, male gender, rurality, large family sizes, lower socio-economic status, and lower maternal education were similar to risk factors associated with fatal drowning events in Bangladesh and other LMICs [1,21,22]. Non-fatal drowning events led to short-term disability in a minority of events, for an average of 2 days. Common difficulties faced after a non-fatal drowning events included an inability to stand for a long period, emotional effects, reduced concentration, and difficulty walking long distances. 

This study showed that disability resulting from non-fatal drowning was relatively uncommon. There are several possible explanations for these results. Firstly, the findings may be an underestimate. Under-reporting of non-fatal drowning and challenges in documenting the associated health burden are well established [23]. The tool was selected based on reviews and expert opinions, but the findings are challenging to interpret given the single point of data collection, which did not track changes in disability over a period of time. In addition, the experience of disability was limited to a time scale of 30 days. Although shorter recall periods may be more accurate than longer recall periods, it is possible that disabilities that occurred within the weeks and months after the non-fatal drowning event were not picked up, as the survey assessed drowning events from the past year. Both temporary and long-term disabilities can still impact mental and physical wellbeing and bring significant burden to families and should be assessed. However, questions for a tool with a longer recall period may require alternate phrasing, as participants would be unlikely to remember exactly how many days they experienced difficulties in the last few months. Such a tool may only be able to measure whether the victim experienced the difficulty or not, and for approximately how long. The trade-off between accuracy and data completeness could be better optimised by a longer recall period in a validated tool. 

Another concern is the validity of this tool for children. Many of the indicators were not appropriate to the highest risk age group (1–14 years old), who are less involved in work/school or community activities. The WHODAS instrument was originally developed for use in adults but has been validated for use in youth as young as 15 years [24]. Furthermore, in the case of children, the WHODAS questionnaire was based on the caregiver’s report, which may have resulted in the under-reporting of symptoms. Other researchers have highlighted the inappropriateness of specific questions in the WHODAS instrument for children and a child version is currently being field-tested, but has not yet been validated [25].

The other reason our study may not have shown substantial disability is that, often, long term neurological impacts in survivors of drowning incidents may not be recognised immediately, or even after a short follow-up period, particularly in young children where cognitive effects are not obvious until later on in life [5]. Several important factors contribute to the extent of the effects of a drowning incidents, including submersion time [26], but these were not assessed in this study. Differentiating between pre-existing disability and any disability arising post-drowning also made it challenging to obtain accurate estimates. 

Another possible explanation for low morbidity found is that drowning victims who suffered serious injuries that would have eventually led to disability may have died instead. Drowning events in the Barishal Division are often not referred to healthcare providers, and appropriate care is not given, potentially leading to greater mortality. Inappropriate post-drowning resuscitation practices commonly used in this region may also reduce the likelihood of survival [21]. Hence, only the least severe drowning cases may result in surviving events.

Other instruments considered to measure disability had their limitations; a study by Suimonen et al. (2011) used the HRQoL instrument to assess long-term neurological outcomes in individuals who experienced a drowning incident during childhood and showed a significant loss in HRQoL in the older age group, but not in children. However, this was done through postal survey at least two years after the incident and was limited to children aged 8 years and older and those who had been resuscitated after the drowning incident [27]. Other child-specific tools such as the Paediatric Quality of Life Inventory [28] might be a more suitable option for future testing, but even this is recommended for children from the age of two years and its appropriateness for low income contexts has been questioned [29].

A systematic review by Solans et al. [30] investigating the availability of generic and disease-specific instruments for measuring the health-related quality of life in children identified several instruments, although most were developed for high-income settings, the lower age limit of self-reported measures was 5–6 years old, and many needed to be administered by clinicians. Instruments that could be integrated into household surveys had similar limitations and were also quite lengthy, including the KIDSCREEN-52 quality of life measure [31]. The drawback of many of these tools is that they are age-specific, and different versions are required for different age groups, which means their use in large population-based surveys is challenging. The new PARADISE tool is a 24-item tool developed to assess impacts on brain disorders and is based on psychosocial difficulties [32]. However, this has also been developed and used in Europe for those aged over 18 years. There is a need, therefore, for the development of a tool to assess both physical and neurological disability in young children from the age of one years old, that can be administered by a lay-person and is validated in a community, including LMIC settings.

Given a dearth of tools that can accurately assess disability in young children and the possible emergence of disabilities years after the event, longitudinal studies may be required to assess the long-term effects of drowning. The development of a tool by WHO for non-fatal drowning in community settings is a welcome development, however it needs to be validated in a LMIC context, particularly among children [33]. The use of registries that track drowning events in Bangladesh may be useful in this regard [34]. However, while tracking drowning deaths may be feasible as such instances would be noticed by community health workers, leaders, and health centres, and are easily identifiable, non-fatal drowning events may remain underreported. Parents would have to be consistently encouraged to report on these events, even if they appear minor and have little visible effect on their children. Data managers for the registries should be trained to assess whether the event can be classified as a non-fatal drowning event as per definition. A register may only be implementable in a few sample communities, given the monitoring and data quality processes involved.

The risk factors of non-fatal drowning were similar to those of fatal drowning [21,22,23]. Interventions for the prevention of non-fatal drowning for this rural, LMIC region would therefore be similar to those aiming to prevent fatal drownings. For children, the WHO recommends the use of home-based barriers to prevent children’s access to water, the provision of supervised childcare services in physically enclosed creches, and swim and rescue training. For adults, regulations for water transport and workplace safety should be enforced. Community members should also be upskilled in first response to drowning [1].

## 5. Conclusions

Our study reported on non-fatal drowning events in Bangladesh. While the majority of survivors in our study did not experience disability, the findings demonstrate the need for better tools to measure drowning-related morbidity and the longer term follow-up of impacts, especially in young children, as these may have been underestimated. Training communities in appropriate post-drowning first response may also lead to a greater proportion of drowning victims surviving but facing morbidity. One fit-for-all approach to measuring disability from non-fatal drowning might not be viable, and thus there is a need to develop and identify methodologies to address some critical gaps in the low-and middle-income context, and child relevant outcome measures, informing the global drowning prevention agenda. The potential for new tools to draw comparison in diverse contexts may be considered.

## Figures and Tables

**Table 1:** Socio-demographic characteristics of people reporting non-fatal drownings (NFD).

Variable	**Level**	**% of All NFDs (95% Confidence Limits)**
Age (years)	0–4	68% (67–70%)
	5–9	28% (27–30%)
	10–17	2% (1–2%)
	18+	2% (1–2%)
Gender	Male	51% (50–53%)
	Female	48% (47–50%)
Setting	Rural	94% (91–96%)
	Urban	6% (4–9%)
Monthly household income (taka/month)	0–7999	17% (15–20%)
	8000–9999	24% (22–27%)
	10,000–11,999	19% (17–20%)
	12,000–15,999	26% (24–28%)
	16,000+	13% (12–15%)
Socioeconomic quintile	1 (lowest)	25% (22–27%)
	2	26% (24–28%)
	3	19% (18–21%)
	4	17% (15–18%)
	5 (highest)	14% (12–15%)

**Table 2:** Non- fatal drowning event rates by age, sex, and rurality in the Barishal Division, Bangladesh.

	Male	Female	Rural	Urban	All Persons
Age (Years)	Rate per 100,000 (95% CI)	Rate per 100,000 (95% CI)	Rate per 100,000 (95% CI)	Rate per 100,000 (95% CI)	Rate per 100,000 (95% CI)
<1 (infant)	116.4 (23.8–209.0)	149.4 (35.5–263.4)	145.3 (58.0–232.6)	32.6 (0.0–94.7)	133.0 (53.2–212.8)
1–4	6124.2 (5416.6–6831.8)	5501.2 (4917.3–6085.1)	6103.2 (5469.2–6737.2)	3377.7 (2495.2–4260.3)	5810.8 (5209.9–6411.7)
5–9	1806.7 (1581.2–2032.2)	1868.2 (1601.0–2135.4)	1905.8 (1672.4–2139.2)	1217.7 (837.0–1598.5)	1837.6 (1614.9–2060.4)
10–14	65.9 (31.1–100.8)	127.9 (79.4–176.3)	97.7 (64.7–130.6)	91.6 (21.2–162.1)	97.1 (65.4–128.8)
15–17	44.6 (13.8–75.4)	19.0 (0.0–39.0)	36.5 (13.5–59.4)	0	32.6 (12.0–53.2)
18–24	38.2 (7.3–69.2)	20.5 (3.6–37.4)	32.6 (13.5–51.7)	0	28.9 (12.0–45.9)
25–39	19.9 (9.0–30.7)	4.8 (0.0–9.6)	13.5 (6.7–20.3)	0.9 (0.0–2.5)	12.1 (6.1–18.1)
40–59	32.1 (13.9–50.3)	5.9 (0.3–11.6)	20.5 (10.1–31.0)	5.8 (0.0–14.1)	18.9 (9.6–28.3)
60+	17.0 (4.4–29.5)	8.7 (0.0–19.0)	12.7 (4.1–21.2)	13.9 (0.0–33.7)	12.8 (5.0–20.6)
Total	728.8 (644.0–813.6)	665.6 (592.7–738.5)	731.9 (653.5–810.2)	405.2 (300.4–510.1)	696.7 (622.0–771.4)

**Table 3:** Impact of disability from drowning incident on ability to carry out daily activities.

WHODAS Questions	Weighted Frequency	Mean (SE)	95% CI
Overall, in the past 30 days, how many days were these difficulties present?	4859	2.4 (0.4)	1.6, 3.2
In the past 30 days, for how many days were you totally unable to carry out your usual activities or work because of any health condition?	4866	2.2 (0.4)	1.4, 3.0
In the past 30 days, not counting the days that you were totally unable, for how many days did you cut back or reduce your usual activities or work because of any health condition?	4702	1.7 (0.3)	1.1, 2,4

**Table 4:** Frequency by 12 domains of WHODAS, post the most recent non-fatal drowning event.

WHODAS Items	No Difficulty	Mild Difficulty	Moderate Difficulty	Severe Difficulty	Extreme Difficulty	Not Applicable
In the Past 30 Days…	*n* (%)	*n* (%)	*n* (%)	*n* (%)	*n* (%)	*n* (%)
Standing for long periods such as 30 min	3626 (72.9)	606 (12.2)	71 (1.4)	19 (0.4)	-	654 (13.1)
Difficulty in taking care of household responsibilities	2177 (43.8)	123 (2.5)	43 (0.9)	4 (0.1)	27 (0.5)	2600 (52.3)
Difficulty in concentrating on doing something for ten minutes	2875 (57.9)	415 (8.4)	108 (2.2)	46 (0.9)	3 (0.1)	1519 (30.6)
Difficulty in joining in community activities (for example, festivities, religious or other activities)	2644 (53.2)	147 (3.0)	45 (0.9)	10 (0.2)	1 (<0.001)	2123 (42.7)
Difficulty in emotionally affected by your health problems	2690 (54.1)	662 (13.3)	186 (3.7)	71 (1.4)	2 (<0.001)	1363 (27.4)
Difficulty in concentrating on doing something for ten minutes	2834 (57.1)	483 (9.7)	92 (1.9)	53 (1.1)	1 (<0.001)	1504 (30.3)
Difficulty in walking a long distance such as a kilometre	2839 (57.1)	462 (9.3)	112 (2.3)	36 (0.7)	1 (<0.001)	1518 (30.6)
Difficulty in washing your whole body	3138 (63.1)	318 (6.4)	55 (1.1)	40 (0.8)	3 (0.1)	1418 (28.5)
Difficulty have you/the injured household member had in getting dressed	3250 (65.5)	201 (4.0)	35 (0.7)	25 (0.5)	3 (0.1)	1450 (29.2)
Difficulty in dealing with people you do not know	2902 (58.4)	314 (6.3)	44 (0.9)	15 (0.3)	1 (<0.001)	1692 (34.1)
Difficulty in maintaining a friendship	2986 (60.1)	357 (7.2)	72 (1.4)	17 (0.3)	2 (<0.001)	1533 (30.9)
Difficulty in your day-to-day work/school	2809 (56.6)	436 (8.8)	161 (3.2)	115 (2.3)	2 (<0.001)	1436 (29.0)

**Table 5:** Factors associated with non-fatal drowning among samples from Barishal Division, Bangladesh.

		Univariate Analyses			Multivariable			
Variable	OR	95% CI		*p*-Value	OR	95% CI		*p*-Value
Gender								
Female	reference				reference			
Male	1.095	1.019	1.177	0.0134	1.079	1.000	1.165	0.0508
Age								
0–4 years	290.7	202.2	418.0	<0.0001	271.5	190.4	387.2	<0.0001
5–9 years	4.1	2.8	6.0		3.7	2.5	5.5	4.149
10–17 years	106.7	75.3	151.4		95.7	68.0	134.9	
18+ years	reference				reference			
Maternal education								
None	1.399	0.962	2.034		1.540	1.064	2.229	
1 to 5 years	1.559	1.103	2.203	<0.0001	1.507	1.073	2.118	0.1293
6 to 8 years	1.862	1.321	2.625		1.557	1.105	2.194	
9 to 12 years	1.586	1.129	2.229		1.437	1.020	2.024	
13 to 17 years	reference				reference			
Wealth quintile								
Q1 (lowest)	2.156	1.829	2.543	<0.0001	1.932	1.624	2.299	<0.0001
Q2	2.172	1.873	2.517		2.031	1.742	2.368	
Q3	1.611	1.368	1.899		1.499	1.271	1.767	
Q4	1.437	1.242	1.662		1.396	1.204	1.617	
Q5 (highest)	reference				reference			
Number of children in household								
1 child	Reference			Reference				
2 children	1.575	1.427	1.739	<0.0001	1.128	1.012	1.257	0.0258
3 children	1.889	1.697	2.102		1.205	1.075	1.351	
4 children	2.256	1.945	2.617		1.256	1.063	1.485	
5+ children	2.461	1.929	3.140		1.217	0.961	1.541	
No children	0.026	0.013	0.053		0.819	0.429	1.562	

## Data Availability

Data are available on reasonable request.
